# The ESX System in *Bacillus subtilis* Mediates Protein Secretion

**DOI:** 10.1371/journal.pone.0096267

**Published:** 2014-05-05

**Authors:** Laura A. Huppert, Talia L. Ramsdell, Michael R. Chase, David A. Sarracino, Sarah M. Fortune, Briana M. Burton

**Affiliations:** 1 Department of Molecular and Cellular Biology, Harvard University, Cambridge, Massachusetts, United States of America; 2 Department of Immunology and Infectious Diseases, Harvard School of Public Health, Boston, Massachusetts, United States of America; 3 Thermo Fisher Scientific, BRIMS Unit, Cambridge, Massachusetts, United States of America; INRA Clermont-Ferrand Research Center, France

## Abstract

Esat-6 protein secretion systems (ESX or Ess) are required for the virulence of several human pathogens, most notably *Mycobacterium tuberculosis* and *Staphylococcus aureus*. These secretion systems are defined by a conserved FtsK/SpoIIIE family ATPase and one or more WXG100 family secreted substrates. Gene clusters coding for ESX systems have been identified amongst many organisms including the highly tractable model system, *Bacillus subtilis*. In this study, we demonstrate that the *B. subtilis yuk*/*yue* locus codes for a nonessential ESX secretion system. We develop a functional secretion assay to demonstrate that each of the locus gene products is specifically required for secretion of the WXG100 virulence factor homolog, YukE. We then employ an unbiased approach to search for additional secreted substrates. By quantitative profiling of culture supernatants, we find that YukE may be the sole substrate that depends on the FtsK/SpoIIIE family ATPase for secretion. We discuss potential functional implications for secretion of a unique substrate.

## Introduction

Bacterial secretion systems play a critical role in the ability of bacterial cells to interface with their environment. In addition to the Sec (secretory) and Tat (twin-arginine translocation) systems that are involved in protein export (i.e. transport across the cytoplasmic membrane) [1]–[3], several outer membrane machineries have been described that complete protein secretion [4]–[7]. These secretion systems are less widely conserved and have more specific functions, such as horizontal gene transfer, nutrient uptake, and enabling virulence [8]. Recent studies identified a novel, dedicated export system called the Esat-6 secretion system (ESX or Ess), which is now known to be present in many bacteria including the archtypical Gram-positive bacterium *Bacillus subtilis* [9]–[12].

ESX protein secretion systems were initially identified in *Mycobacterium tuberculosis*, where it was demonstrated that the ESX-1 secretion system is responsible for the export of the small proteins ESAT-6 and CFP-10 (also named EsxA and EsxB respectively)[13], [14]. EsxA is a 100-amino acid peptide that lacks an N-terminal signal sequence and has a helix-turn-helix structure with a WXG motif in the central turn, so it is also known as a WXG100 protein [11]. Bioinformatic studies using *in silico* methods to search for WXG100 family genes in other bacterial species have predicted the existence of ESX secretion systems in other Actinobacteria, some Firmicutes, and several Chloroflexi [11], [12], [15]. These predictions have been validated in several species, including *Staphylococcus aureus* [16]–[19], *Bacillus anthracis* [20], and *Streptomyces coelicolor* [21]. Intriguingly, genes homologous to some ESX components are sporadically distributed more broadly, including among the Proteobacteria [15]. ESX secretion systems are now defined by the presence of one or more WXG100 family substrates in addition to an FtsK/SpoIIIE family ATPase, often called EccC/EssC, that is required for substrate secretion [10].

The primary function of the proteins exported by ESX secretion systems remains unknown and therefore it is unclear whether the ESX systems share a conserved function(s). Numerous studies have demonstrated that the *M*. *tuberculosis* ESX-1 secretion system is essential for the virulence of this human pathogen; some studies suggest that the ESX-1 substrates compromise the integrity of the phagosomal membranes during macrophage infection [22]–[25], while other work suggests that the ESX secreted substrates are important for bacterial cell wall maintenance [23], [26], [27]. In addition, several of the recently identified ESX systems play a role in bacterial pathogenesis, including the ESX systems in *S. aureus* and *B. anthracis* [16]–[20], [28]. However, there are also examples of ESX systems that do not play a role in virulence, such as the ESX system in the plant pathogen *Streptomyces scabies* that modulates sporulation and development [29]. Furthermore, ESX systems are predicted in non-pathogenic bacteria, and such systems have been validated in the soil bacterium *S. coelicolor* [11], [21] and in *M. smegmatis* [30].

**Figure 1:**
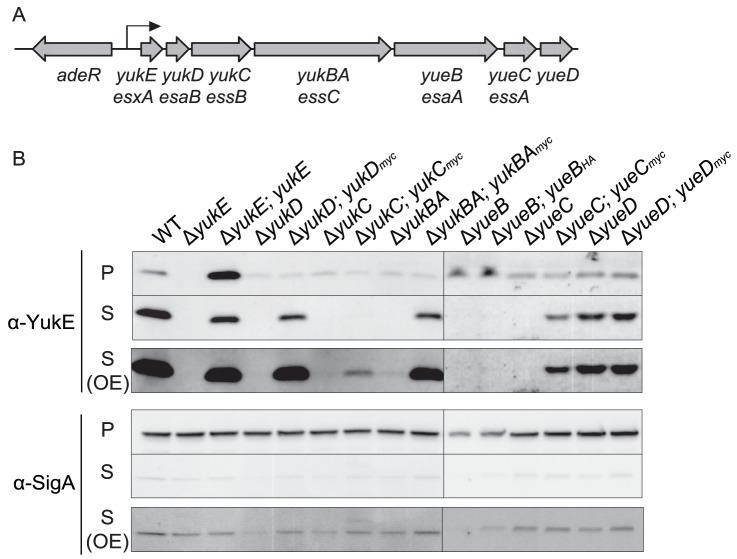
YukE is secreted, and secretion of YukE depends on other proteins encoded by the *yuk*/*yue* locus. A: Schematic depicting the *yuk*/*yue* locus and surrounding genes. Currently, there are five annotated genes in the *yuk* operon: *yukE, yukD, yukC, yukBA*, and *yueB* [31], [32]. Recent high throughput transcriptomics data implicates *yueC* and/or *yueD* as potential members of the *yuk*/*yue* locus as well [33]. The predicted promoter (*Pyuk*) is indicated with an arrow. Homology to genes of other ESX/Ess systems is indicated below the corresponding *yuk*/*yue* gene name. B: Secretion assay for YukE. Cells were grown in LB medium to OD600nm of approximately 1.0–1.3. The cell pellet (P) was separated from the culture supernatant (S) by centrifugation. The pellet fractions were prepared into whole cell lysates and the supernatant fractions were filtered through a 0.2 micron filter and TCA precipitated. Samples were analyzed by SDS-PAGE under reducing conditions and immunoblot analysis with an α-YukE antibody and an α-SigmaA antibody as a loading/lysis control. The supernatants are shown in two exposures; the overexposed α-YukE blot (OE) allows visualization of faint bands. Data are representative of at least three biologically independent experiments. Pellet samples are equivalent to 0.1 OD and twenty-fold more was loaded for supernatant samples. Equivalent loading of precipitated supernatant samples was confirmed by densitometry of the Coomassie-stained gel.

Bioinformatic analysis predicted that the *yuk* operon in the non-pathogenic bacterium *Bacillus subtilis* may encode an ESX protein secretion system [11]. Currently, there are five annotated genes in the *yuk* operon: *yukE*, *yukD*, *yukC*, *yukBA*, and *yueB* [31], [32] (Figure 1A). The current annotation of the *yuk* operon suggests a terminator after *yueB*, but recent high throughput transcriptomics data implicates *yueC* and/or *yueD* as potential members of the *yuk*/*yue* locus as well [33]. By sequence analysis, the signature ESX/Ess proteins are represented in this system: YukE is homologous to the secreted virulence factor EsxA in *M. tuberculosis* and YukBA is predicted to be an FtsK/SpoIIIE family ATPase homologous to EccCa and EccCb in *M. tuberculosis* and EssC in *S. aureus* [11], [16].

In this study, we demonstrate that the *yuk*/*yue* locus in *B. subtilis* encodes functional components of an ESX protein secretion system. We demonstrate that the small WXG100 protein, YukE, is secreted from cells. The secretion of YukE depends upon the other gene products encoded by the locus, including the other signature member of ESX secretion systems, the FtsK/SpoIIIE family ATPase YukBA. These results confirm a recent study of the *yuk*/*yue* locus components [34], and expand on that work by establishing the specificity of each of the locus components. Using an unbiased mass spectrometry approach, we find YukE to be the only measurable YukBA-dependent substrate. Further, we demonstrate that the presence of the locus and the constitutive secretion of YukE provide neither a growth disadvantage nor a competitive advantage for the strain.

## Results

### The Bacillus subtilis yuk/yue locus encodes a secreted protein, YukE

All ESX protein secretion systems that have been studied to date have been shown to secrete at least one WXG100 family protein homologous to the prototypic ESX-1 substrate EsxA [13], [16], [20], [21]. In *B. subtilis*, this protein is encoded by *yukE*. Therefore, our first experimental objective was to determine whether YukE is secreted from the *B. subtilis* cell. To address this question, we grew cultures of the wild-type domesticated strain of *B. subtilis* (PY79) in nutrient-rich LB medium to mid-exponential phase, harvested whole cell pellets, and filtered the culture supernatants. Proteins in the culture supernatant were concentrated by TCA precipitation and analyzed by SDS-PAGE. Presence of YukE was assessed using a primary antibody raised against recombinant full-length YukE. As a lysis control, we tested for the presence of the cytosolic protein RNA polymerase sigma factor SigmaA by immunoblotting with α-SigmaA antibodies [35]. In these experiments, we detected YukE in both the pellet and supernatant fractions (Figure 1B). These data confirm the prediction and recent demonstration that YukE is secreted from the cell [34]. In contrast to the previous work, we were able to detect YukE secretion in a domesticated laboratory strain. We found that YukE was secreted in all conditions tested, ranging from growth in nutrient-rich media to the nutrient-limiting conditions that promote competence and biofilm formation (Figure S1).

### YukE secretion depends upon other yuk/yue locus components

Next, we asked whether YukE secretion depends upon the other gene products in the *yuk*/*yue* locus. To address this question, we created a series of *yuk*/*yue* knockout strains. Each *yuk*/*yue* gene was individually replaced with an antibiotic resistance cassette and the *yuk* promoter (*Pyuk*) was reinserted after the resistance cassette to drive expression of the downstream operon genes. We used the intergenic region between *yukE* and *adeR* as the *yuk* promoter, and confirmed that *Pyuk* was transcriptionally active by inserting a *Pyuk-lacZ* construct at an ectopic integration site (*amyE::Pyuk-lacZ*) and assessing transcriptional activity. The β-galactosidase activity in this strain was approximately three-fold lower than the β-galactosidase activity in a strain with *lacZ* integrated at the endogenous *yuk* operon start site (Ω*Pyuk-lacZ*) (Figure S2). This was ultimately useful, because genome-wide expression studies indicate that *yukE* expression is at least twice as high as the expression of other *yuk* operon genes [36]. Therefore, we reasoned that using our weaker *Pyuk* should result in approximately wild-type levels of transcription of the downstream genes. We confirmed that the reinserted *Pyuk* drove expression of downstream *yuk* genes, although resulting protein levels were approximately two-fold higher than native levels, as assessed by semi-quantitative immunoblotting (Figure S2).

To determine whether the genes of the *yuk*/*yue* locus are required for YukE secretion, we tested whether YukE is produced and secreted in each of the *yuk*/*yue* knockout strains. Currently, there are five annotated genes in the *yuk* operon: *yukE*, *yukD*, *yukC*, *yukBA*, and *yueB* [31], [32]. Knocking out each gene in the annotated *yuk* operon (*yukE*-*yueB*) individually abolished YukE secretion in all five of these strains (Figure 1B). Recently, transcriptomic profiling has implicated *yueC* and/or *yueD* as potential members of the *yuk*/*yue* operon as well [33]. Therefore, we also tested whether YukE is secreted in Δ*yueC* and Δ*yueD* strains. YukE was not secreted in the Δ*yueC* strain, demonstrating that YueC is required for YukE export, but it was secreted in the Δ*yueD* strain, suggesting that YueD is not required for YukE export (Figure 1B).

To demonstrate the specificity of these results, we constructed complementation strains by inserting the corresponding *yuk*/*yue* gene at an ectopic integration site under the control of an inducible promoter. We attached a C-terminal Myc or HA tag to each of the complementation constructs (except for the untagged YukE complementation construct), thereby allowing us to verify presence of the complementing protein by immunoblot (Figure S3). YukE secretion was restored to wild-type levels in the Δ*yukD,* Δ*yukBA*, and Δ*yueC* strains upon expression of *yukD-myc, yukBA-myc*, and *yueC-myc* respectively (Figure 1B). Densitometric analysis of secretion levels in each strain is presented in Table 1; values indicate the percentage of total YukE in each strain that is localized to the pellet versus culture supernatant. Complementation of Δ*yukC* with *yukC-myc* did not restore YukE secretion to wild-type levels, but partial restoration of YukE secretion can be seen in an overexposed blot (Figure 1B). We were unable to complement YukE secretion in the Δ*yueB* strain, despite attempts with untagged and several tagged versions of YueB. Nonetheless, YukE secretion appears dependent upon the *yueB* gene product and a recent study produced a complementing construct which confirms the specificity of a *yueB* deletion [34]. Thus we conclude that YukE secretion requires the full *yuk* operon as well as *yueC*, but not *yueD*.

**Table 1:** Quantification of secreted YukE.

STRAIN	% SigA in pellet	% SigA in supernatant	% YukE in pellet	% YukE in supernatant
Wildtype	99.97	0.03	81.06	18.94
Δ*yukE*	99.99	0.01	N/A	N/A
Δ*yukE; yukE*	100.00	0.00	97.19	2.81
Δ*yukD*	100.00	0.00	100.00	0.00
Δ*yukD; yukD-myc*	99.99	0.01	65.20	34.80
Δ*yukC*	99.99	0.01	100.00	0.00
Δ*yukC; yukC-myc*	99.98	0.02	99.65	0.35
Δ*yukBA*	99.98	0.02	100.00	0.00
Δ*yukBA; yukBA-myc*	99.98	0.02	78.44	21.56
Δ*yueB*	99.94	0.06	99.49	0.51
Δ*yueB; yueB-HA*	99.84	0.16	99.67	0.33
Δ*yueC*	99.74	0.26	100.00	0.00
Δ*yueC; yueC-myc*	99.77	0.23	88.41	11.59
Δ*yueD*	99.86	0.14	87.15	12.85
Δ*yueD; yueD-myc*	99.79	0.21	87.97	12.03

Densitometric analysis of the YukE and SigmaA proteins from the blots shown in Figure 1.

The divergently transcribed gene *adeR* (formerly annotated as *yukF*) is a predicted transcription factor. Since regulatory proteins are often coded in the general vicinity of the genes they regulate, we also tested for YukE secretion in an *adeR* knockout strain, and found that YukE was still secreted in this background (Figure S4). This result is consistent with the idea that *yuk*/*yue* activity is perhaps principally regulated through stress response pathways including those governed by DegS/U and Spo0A [33], [34], [37]–[40], although inputs from other regulatory pathways may remain to be discovered.

### YukE is the only protein detected to be dependent upon YukBA for secretion

To gain insight into possible function(s) of the *yuk*/*yue* system, we next sought to determine whether there are additional secreted proteins dependent upon the *yuk*/*yue* locus for secretion. Besides YukE, there is one other predicted WXG100 protein encoded in the *B. subtilis* genome, YfjA, and therefore this protein was a candidate *yuk*/*yue* substrate. [11]. In addition, secretion of LXG-motif proteins and non-WXG100 proteins has been reported in other ESX secretion systems, and these proteins are often encoded away from the primary ESX/Ess locus [20], [41]. Therefore, we decided to use an unbiased, quantitative proteomics approach to analyze the full profile of *yuk*/*yue*-dependent proteins in the culture supernatant.

**Figure 2:**
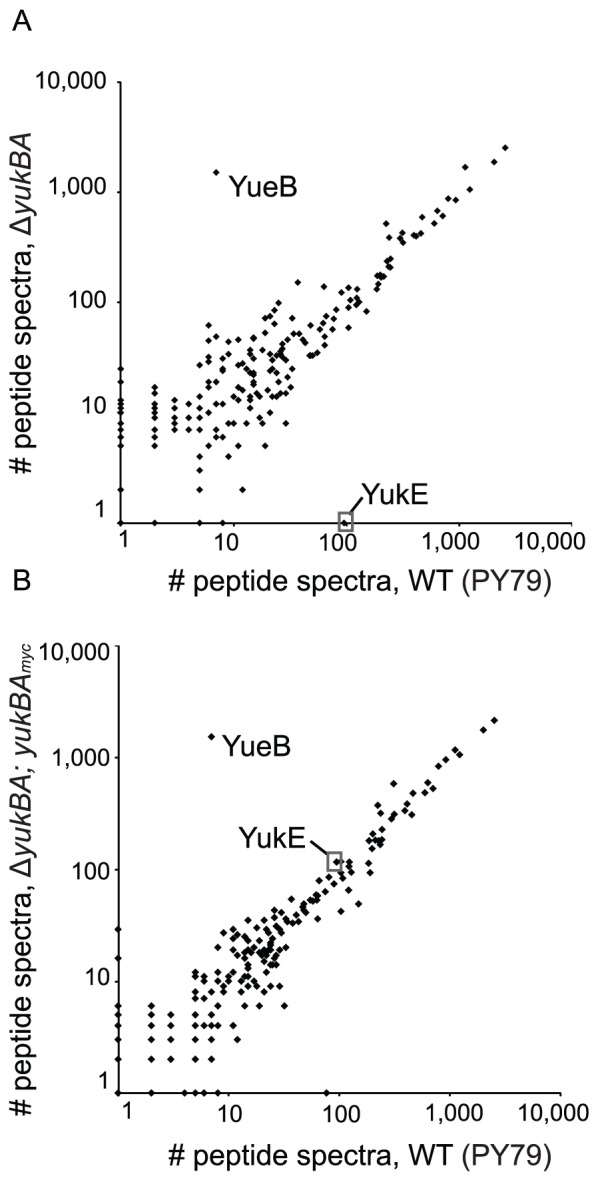
YukE is the only protein dependent upon YukBA for secretion. (A). and (B). The relative abundance of proteins detected in the culture supernatant of the wild-type strain (PY79) versus the Δ*yukBA* strain (A) or the complemented Δ*yukBA*; *yukBA-myc* strain (B). Cells were grown in nutrient-limiting 1XMC medium to mid-exponential phase, and the supernatant fractions were filtered through a 0.2 micron filter and TCA precipitated. The proteins in the culture supernatant were analyzed by mass spectrometry. Protein abundance was determined by spectral count analysis; spectral count data are combined totals from three biologically independent samples for each strain. Where no spectra were identified, an arbitrary value of 1 was assigned. The data point for YukE is circled in each graph. The point for YukE is at (95,1) in Figure 2A and at (95, 116) in Figure 2B. The complementation strain was constructed with the ectopically expressed *yukBA* gene disrupting the native *amyE* locus. Thus, as expected, AmyE peptides are underrepresented in the complementation strain as compared to both wild-type and Δ*yukBA* strains; the point located at (77, 1) in Figure 2B corresponds to the peptides assigned to AmyE. High levels of YueB peptides in the Δ*yukBA* and complement strains is a consequence of strain design; the *yuk* promoter was reinserted after the *yukBA* deletion to drive expression of the downstream genes.

In addition to the virulence factor polypeptides, the FtsK/SpoIIIE family ATPases are a signature of ESX loci. Thus, using quantitative mass spectrometry, we compared the proteins in culture supernatants of the wild-type domesticated strain and the ATPase deletion strain Δ*yukBA* grown in defined media. Consistent with our immunoblot assay, we detected YukE in the supernatant of the wild-type strain in a manner that was dependent upon *yukBA* (Figure 2A, 2B). YukE secretion was restored in the YukBA complementation strain (Figure 2B). Ninety-five YukE-specific peptide spectra were detected in the supernatant from the wild-type strain, no peptides were detected in the Δ*yukBA* strain and 116 YukE-specific peptide spectra were detected in the Δ*yukBA*; *yukBA-myc* complementation strain. We detected high levels of YueB peptides in the culture supernatant of the Δ*yukBA* and complement strains (Figure 2A, 2B), which is an expected consequence of the strain design. Briefly, the *yuk* promoter was reinserted after the *yukBA* deletion to drive expression of the downstream genes, as otherwise this would be a polar mutation. Most surprisingly, we did not detect any other proteins with the same secretion profile as YukE in these conditions. Therefore, by this method and under these growth conditions, we found YukE to be the only protein that requires the ATPase YukBA for secretion.

### The yuk/yue locus does not confer a growth or competition phenotype

**Figure 3:**
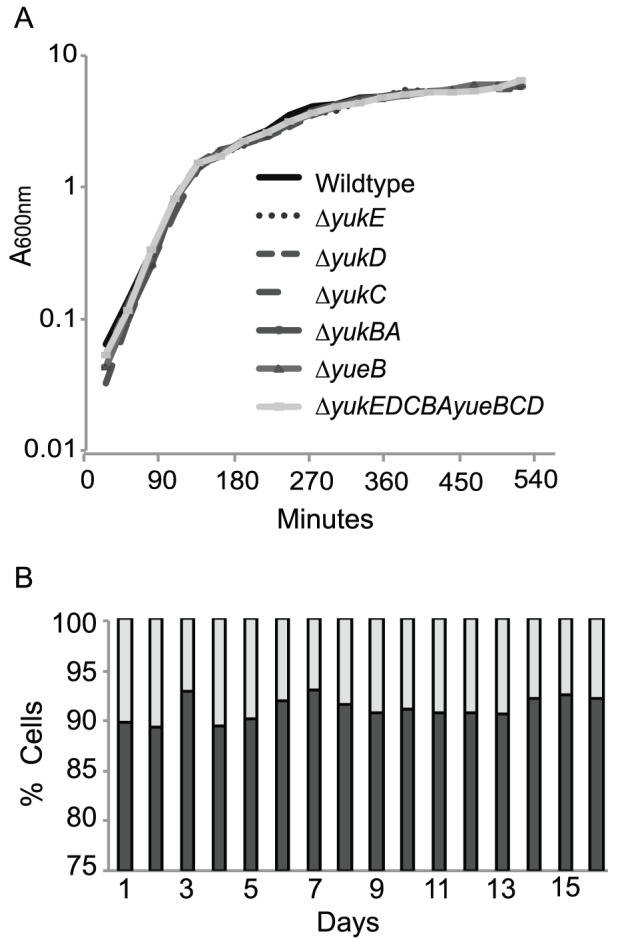
*yuk*/*yue* knockout strains do not have a growth or competition defect compared to the wild-type strain. A: Growth curve of the wild-type strain (PY79) and *yuk*/*yue* knockout strains grown in LB medium shaking at 37°C. The OD600nm was taken every 30 minutes for a total of 540 minutes. The following *yuk*/*yue* knockout strains were tested: Δ*yukE*, Δ*yukD*, Δ*yukC*, Δ*yukBA*, Δ*yueB*, and Δ*yukEDCBAyueBCD*. B: The results of a representative competition experiment between Δ*yukEDCBA* (light gray) versus the wild-type reporter strain (dark gray) in nutrient-rich LB medium. This competition had a starting ratio of 10% Δ*yukEDCBA* cells to 90% wild-type cells. The percentages were determined by counting the number of blue and white colonies on a single plate each day (typically 150–250 colonies per plate) and then calculating the percentage of colonies from each strain. Shown are the mean percentages averaged from triplicate platings for each day.

The biological function of the *yuk*/*yue* locus remains unknown but it is highly unusual for a secretion system to have only a single substrate. Further, since all conditions we tested yielded secreted YukE, we speculated that the *yuk*/*yue* knockout strains might display a growth or competition phenotype. We first tested whether various *yuk*/*yue* knockout strains have a growth defect compared to the wild-type domesticated strain by conducting growth assays. The growth curves of the *yuk*/*yue* knockout strains were statistically indistinguishable from the growth curve of the wild-type domesticated strain, indicating that the *yuk*/*yue* knockout strains do not have a growth defect under standard, nutrient-rich laboratory conditions (Figure 3A). Next, we performed competition assays between the wild-type domesticated strain and *yuk*/*yue* knockout strains. We found that the *yuk*/*yue* knockout strains did not have a statistically significant competitive advantage or disadvantage compared to the wild-type domesticated strain in nutrient-rich or nutrient-limiting media (Figure 3B and Figure S5).

## Discussion

Here, we have confirmed that the WXG100 protein, YukE, is a secreted protein, as predicted by its homology to the secreted virulence factor EsxA of *M. tuberculosis* and EsxA of *S. aureus*. YukE secretion is dependent upon each of the four other genes encoded within the annotated *yuk* operon as well as *yueC*, and we have confirmed the specificity of these dependencies by complementation. Most notably, secretion of YukE depends on the conserved FtsK/SpoIIIE family ATPase YukBA, the other signature member of ESX secretion systems. Furthermore, YukE secretion depends on YukD and YukC, which are homologous to proteins EsaB and EssB respectively in the Ess secretion system of *S. aureus*. Together with another recent study, these results suggest that the *yuk*/*yue* locus in *B. subtilis* encodes a *bona fide* ESX protein secretion system [34]. The predicted topologies and subcellular localizations of the Yuk/Yue proteins suggest a membrane-bound secretion complex. Indeed, the envelope protein YueB has been implicated as a phage receptor (28), but this information has yet to provide additional clues as to the complete architecture of the system.

We have found YukE to be the only dedicated substrate of this secretion system thus far; we detected the other predicted WXG100 protein, YfjA, to be equally secreted in all strains tested, suggesting that it is not a YukBA-dependent substrate. Further profiling studies with different strain backgrounds or under different conditions may yet reveal additional substrates. For example, a recent study also detected YukE as a secreted product, although that report suggested that the strain background affects the conditions under which secreted YukE is detected [34].

ESX protein secretion systems are conserved throughout pathogenic and non-pathogenic species. It is currently unclear what the primary function of these systems is and whether ESX secretion systems share a conserved function(s). All ESX systems studied to date have been shown to be responsible for the secretion of a conserved EsxA-like protein substrate [13], [16], [20], [21]; however, these proteins do not have an obvious effector function, and it is unclear how the secretion of a single conserved substrate could be beneficial to bacterial species representing such a wide range of lifestyles and environmental niches.

In *M. tuberculosis*, the ESX-1 system is required for pathogenesis [22]-[24] and several secreted substrates have been identified [13], [14], [41]–[45], but the specific functions of the secreted proteins are unknown. The prevailing hypothesis is that the secreted protein EsxA acts as a pore-forming toxin and induces damage to host cell membranes [22], [25]. *B. subtilis* is not a human pathogen, but it likely encounters eukaryotes in its natural environment so it may similarly play a role in bacterial-eukaryotic interactions. For example, other *B. subtilis* systems have been demonstrated to have anti-nematodal and anti-fungal properties [46], [47], so the Yuk/Yue proteins may have a similar function. Alternatively, components of the ESX systems have been implicated in DNA transfer in both mycobacterial species and in *B subtilis* [48], [49] so the *yuk*/*yue* system may play a role in bacterial-environmental interactions by aiding with competence and DNA transfer.

An alternative hypothesis is that the ESX secreted proteins are required for a housekeeping function such as the maintenance of the bacterial cell wall [23], [26], [27]. In our study, we detect secretion of YukE under all tested conditions so it is possible that YukE is constitutively secreted to provide a function required for cell wall integrity or maintenance. It remains formally possible that YukE is in fact a component of the secretion apparatus itself. Further studies are needed to evaluate these hypotheses.

In this study, we find that YukE is the only identified substrate that is secreted under the conditions we tested. We also find that the *yuk*/*yue* system is not essential under these conditions. Therefore, it is possible that in response to some other stimulus, additional substrates will be identified and the *yuk*/*yue* system may be essential for bacterial growth or survival. This notion is further supported by a few lines of evidence that link regulation of the *yuk*/*yue* locus to the cell's stress response systems. A recent study implicated the two-component DegUS system in regulating YukE secretion, and numerous studies have pointed to the role of the master regulator Spo0A in upregulating *yuk*/*yue* genes [33], [34], [37]–[40]. Together these studies suggest that further work with undomesticated strains may ultimately yield vital clues to the biological role of the *B. subtilis* ESX machinery.

## Materials and Methods

### Strain construction

General methods for molecular cloning and strain construction were performed according to published protocols [50]. Chromosomal DNA isolated from the prototrophic domesticated strain PY79 was used as a template for all PCR amplification. Introduction of DNA into PY79 derivatives was conducted by transformation [51]. The bacterial strains used in this study are listed in Table 2. Complete strain construction information including oligonucleotide primers is included in Supporting Information.

**Table 2:** Strains used in this study.

Strain	Genotype	Source, Reference
PY79	Prototrophic domesticated laboratory strain	[56]
bLH015	*yukE::erm-Pyuk*	This work
bLH018	*yukEDCBA::erm-Pyuk*	This work
bLH019	*amyE::Pyuk-lacZ (spec)*	This work
bLH021	*ΩPyuk-lacZ (cat)*	This work
bLH027	*amyE::Phyperspank-lacZ (spec)*	RL2508 (Gift of Losick Lab)
bLH049	*amyE::kan*	pER82 (Gift of Rudner Lab)
bLH078	*adeR::erm; amyE::Pyuk-lacZ (spec)*	This work
bLH107	*yukEDCBAyueB::erm*	This work
bLH110	*yukBA::erm-Pyuk*	This work
bLH404	*yukBA::erm-Pyuk; amyE::Phyperspank-yukBA-myc (spec)*	This work
bLH421	*yukD::erm-Pyuk*	This work
bLH422	*yukC::erm-Pyuk*	This work
bLH458	*yukD::erm-Pyuk; amyE::Phyperspank-yukD-myc (spec)*	This work
bLH500	*yukC::erm-Pyuk; amyE::Phyperspank-yukC-myc (spec)*	This work
bLH533	*yukE::erm-Pyuk; amyE::Phyperspank-yukE (spec)*	This work
bLH579	*yueB::erm-Pyuk*	This work
bLH581	*yueC::erm-Pyuk*	This work
bLH585	*yueD::erm*	This work
bLH589	*yueB::erm-Pyuk; amyE::Phyperspank-yueB-HA (spec)*	This work
bLH590	*yueB::erm-Pyuk; amyE::Phyperspank-yueB (spec)*	This work
bLH591	*yueC::erm-Pyuk; amyE::Phyperspank-yueC-myc (spec)*	This work
bLH593	*yueD::erm; amyE::Phyperspank-yueD-myc (spec)*	This work

### Media and growth conditions

For general propagation, *B. subtilis* strains were grown at 37°C in LB (lysogeny broth) [52] (10 g tryptone per liter, 5 g yeast extract per liter, 5 g NaCl per liter) or on LB plates containing 1.5% Bacto agar. Where indicated, *B. subtilis* strains were grown in the nutrient-limiting medium *B. subtilis* Medium for Competence (1XMC) [53]. When appropriate, antibiotics were included in the growth medium as follows: 100 µg mL^−1^ spectinomycin, 5 µg mL^−1^ chloramphenicol, 5 µg mL^−1^ kanamycin, 10 µg mL^−1^ tetracycline, and 1 µg mL^−1^ erythromycin plus 25 µg mL^−1^ lincomycin (mls). When required, 100 µM IPTG (isopropyl-β-D-thiogalactopyranoside) was added to cultures or solid media to induce protein expression.

### Bacillus lysates and TCA precipitation

Bacterial strains were grown in LB medium to an OD_600_ of approximately 1.0–1.3. The cells were pelleted and the supernatant was collected. The pellet samples were processed to make whole cell lysates according to standard protocols [53]. Briefly, one milliliter of cells was harvested, lysed in the presence of lysozyme and then boiled for 15 minutes in 1× sample buffer (4% SDS, 250 mM Tris pH 6.8, 20% glycerol, 10 mM EDTA, 1% bromophenol blue, 10% β-mercaptoethanol (BME)). The culture supernatant samples were first filtered through a 0.2 micron filter and then incubated in 10% tricholoracetic acid (TCA) for 12–15 hours at 4°C. The following day, the samples were spun at 15,000xg for 20 minutes to pellet the precipitated proteins, the liquid was poured off, and the pellets were washed with ice-cold acetone. The pellets were resuspended in 100 µL of 1× sample buffer and the samples were boiled for 15 minutes. After processing the pellet and supernatant samples, the proteins were separated by SDS-polyacrylamide gel electrophoresis (SDS-PAGE) and analyzed by immunoblot analysis with appropriate antibodies. Pellet samples are equivalent to 0.1 OD units and twenty-fold more was loaded for supernatant samples. Precipitated supernatant samples were normalized based on Coomassie staining.

### YukE polyclonal antibody generation

A hexahistidine-tagged version of YukE was utilized for antibody production. YukE was PCR-amplified with primers oLH067 and oLH068 using genomic DNA from the wild-type domesticated strain PY79 as a template. The sequence was inserted into an inducible *E. coli* expression vector to make pLH054, which was then transformed into *E. coli* BL21 cells. The cells were induced and YukE was purified from the *E. coli* extracts by nickel-affinity chromatography. Finally, a rabbit polyclonal serum was raised against this protein (Covance).

### Immunoblot analysis

Proteins were separated by SDS-PAGE and transferred to nitrocellulose membrane. The membrane was probed with affinity-purified α-YukE (polyclonal), α-GFP (polyclonal), α-Myc (Novus Biologicals), and/or α-SigmaA (polyclonal) antibodies. Primary antibodies were diluted 1∶1000 (α-YukE), 1∶5,000 (α -GFP), 1∶10,000 (α-Myc) or 1∶1,000,000 (α-SigmaA) in 5% nonfat milk in TBS-0.05% Tween20. The primary antibody was detected using horseradish peroxidase-conjugated goat, α-rabbit immunoglobulin G (Bio-Rad or Jackson Laboratories). Supersignal West Femto chemiluminescent substrate (Thermo Scientific) was used to create a visible chemical reaction. The blots were imaged and densitometric quantitation of YukE secretion was performed using a FlourChem FC2 gel documentation system (Alpha Innotech) and provided software. The densitometry values in Table 1 indicate the proportion of total YukE in each strain that is localized to the pellet versus supernatant; values reflect normalization based on loading of an equivalent of 0.1 OD unit for pellet samples and twenty-fold more sample loaded for supernatant samples.

### Mass spectrometry

Bacterial strains were grown in MC media to an OD_600_ of ∼2.0. The cells were pelleted and the supernatant was collected and filtered through a 0.2 micron filter. Total proteins in the supernatant were obtained by TCA precipitating 30 mL of sample as described above. The samples were prepared for mass spectrometry analysis as described previously [27]. Briefly, samples were separated by molecular weight on a 10–20% Tricine gel (Invitrogen), each lane of the gel was sectioned into 10 roughly equal sized segments, followed by in-gel reduction, alkylation and trypsin digestion. Samples were run on a Thermo Fisher Scientific LTQ Veloz Mass Spectrometer (Thermo Fisher Scientific, Cambridge, MA). Samples were injected onto a Proxeon Easy nLC system configured with a 5 cm×100 µm trap packed with 15–20 µm PS-DVB 300A media, and a 25 cm×100 µm ID resolving column packed with 200A C18AQ media. Buffer A was 96% water, 4% methanol, and 0.2% formic acid. Buffer B was 10% water, 10% isopropanol, 80% acetonitrile, and 0.2% formic acid; loading buffer (sample loading/rinsing buffer) was 96% water, 4% methanol, and 0.2% formic acid. Samples were loaded at 5 µL min^−1^ for 9 min, and a gradient from 0–60% B at 375 nL min^−1^ was run over 70 min, for a total run time of 115 min (including regeneration and sample loading). Injection standards (Michrom Medium Molecule test mix, 5 angios, and the TP4 peptides) were injected at 61 fmoles per sample. Velos was run in a data dependent 15 configuration, with a full scan run in the in enhance scan mode (3^e^4 target), with up to 15MS2 events. Rejection of +1 ions was used in precursor ion selection.

Resulting spectra were searched against a composite database which contained the predicted open reading frames annotated in the genome of *Bacillus subtilis* 168 supplemented with common contaminates using SEQUEST (Thermo Scientific, San Jose, CA). Peptides were filtered at a 1% FDR with PeptideProphet and grouped into proteins with ProteinProphet [54] with a cutoff of 0.95. Spectral counts across the gel slices for three biological replicates were pooled, and then levels of protein abundance between strains were compared using an extended G-test [55]. Data was corrected for multiple testing (Benjamini and Hochberg) using a p value of ≤0.01; for a given protein, a criterion of having ≥5 peptides in at least one strain was set.

## Supporting Information

Figure S1 **YukE is secreted in LB, MC, and MSGG media.** Secretion assays were performed to test YukE secretion from the domesticated PY79 laboratory strain under nutrient-rich growth conditions (LB medium) and nutrient-limiting growth conditions that promote competence (MC medium) or biofilm production (MSGG medium). Cells were grown in LB, MC, or MSGG medium to OD600nm of approximately 1.0–1.3. The cell pellet was separated from the culture supernatant (S) by centrifugation. Supernatant fractions were filtered through a 0.2 micron filter, TCA precipitated, and secretion was analyzed by SDS-PAGE under reducing conditions and immunoblot analysis with an α-YukE antibody and an α-SigmaA antibody as a loading/lysis control.
(EPS)

Figure S2 ***yuk*** **knockout strain schematic and** ***Pyuk*** **promoter activity.** A: Expression from the *yuk* promoter (*Pyuk*) was measured using *Pyuk-lacZ* transcriptional fusions. Two *Pyuk-lacZ* transcriptional fusion reporter strains were used: Ω*Pyuk-lacZ* and *amyE::Pyuk-lacZ*. Because the *yuk* promoter has not been previously characterized, we used the intergenic region between *yukE* and *adeR* as the *yuk* promoter for the latter construct. Strains were grown in LB medium to mid-exponential phase, and then transcriptional activity from P*yuk* was monitored by quantitative β-galactosidase assays. Shown are the mean ± SE of measurements from three independent experiments. B: Schematic showing the native *yuk* operon (top panel with white background) and the *yuk* knockout strains constructed by double crossover recombination (bottom panel with grey background). The *yuk* knockout strains used throughout this work include: Δ*yukE*, Δ*yukD*, Δ*yukC*, Δ*yukBA*, Δ*yueB*, and Δ*yueC*. The predicted *yuk* promoter (*Pyuk*) is indicated with a black arrow, the predicted terminator is indicated with a circle, and *erm* is an antibiotic resistance cassette. *Pyuk* is inserted after the antibiotic resistance cassette to drive expression of downstream genes in the Δ*yukE*, Δ*yukD*, Δ*yukC*, Δ*yukBA*, Δ*yueB* and Δ*yueC* strains. We confirmed that the re-inserted *Pyuk* drives expression of downstream *yuk* genes by inserting Ω*yueB-gfp* into each of these strains and assessing protein levels by semi-quantitative immunoblot with an α-GFP antibody. Compared to YueB-GFP levels detected in the wild-type background (+), YueB-GFP levels in the knockout strains were approximately two-fold higher than native levels (++).
(EPS)

Figure S3 **Expression of epitope-tagged complementing constructs.** Complementation strains were constructed by inserting each corresponding *yuk*/*yue* gene at an ectopic integration site (*amyE*) under the control of an inducible promoter. Immunoblot analysis with α–Myc (YukB-Myc, YukC-Myc, YukBA-Myc, YueC-Myc, YueD-Myc) or α-HA (YueB-HA) antibodies was used to verify the expression of each complementing protein. Astrisks indicate the protein-specific band for each full-length protein. Predicted molecular weight for each protein is as follows: *yukD*, 9 kDa; *yukC*, 52 kDa; *yukBA*, 171 kDa; *yueB*, 120 kDa; *yueC*, 16 kDa; *yueD*, 26 kDa.
(EPS)

Figure S4 **YukE is secreted in an** ***adeR*** **knockout strain.** Secretion assays were performed to test YukE secretion in a wildtype and *adeR* knockout background (bLH078). Cells were grown in LB medium to OD600nm of approximately 1.0–1.3. The cell pellet (P) was separated from the culture supernatant (S) by centrifugation. Supernatant fractions were filtered through a 0.2 micron filter, TCA precipitated, and secretion was analyzed by SDS-PAGE under reducing conditions and immunoblot analysis with an α-YukE antibody and an α-SigmaA antibody as a loading/lysis control. Deletion of *adeR* may have affected the *yuk* operon promoter, possibly causing reduced levels of intracellular YukE in the Δ*adeR* strain as compared to PY79.
(EPS)

Figure S5 **The** ***yukBA*** **knockout strain does not have a competition defect compared to the wild-type strain in MC media.** The results of a representative competition experiment between Δ*yukBA* (light gray) versus the wild-type reporter strain (dark gray) in Media for Competence (MC). This competition had a starting ratio of 90% wildtype cells to 10% Δ*yukBA* cells. The percentages were determined by counting the number of blue and white colonies on a single plate each day (typically 150–250 colonies per plate) and then calculating the percentage of colonies from each strain. Shown are the mean percentages averaged from triplicate platings for each day.
(EPS)

Table S1 **Strains used in this study.**
(DOCX)

Table S2 **Oligos used in this study.**
(DOCX)

Text S1 (DOCX)
