# Supplementary material for: The ESX System in Bacillus subtilis Mediates Protein Secretion
Source: PLoS One. 2014 May 5;9(5):e96267. doi: 10.1371/journal.pone.0096267 (PMC4010439; doi:10.1371/journal.pone.0096267)
Supplement: Table S1 — Strains used in this study. (DOCX) [file pone.0096267.s006.docx]

**Table S1.** Strains used in this study

| **Strain** | **Genotype** | **Source, Reference** |
| --- | --- | --- |
| PY79 | Prototrophic domesticated laboratory strain | ([45](#_ENREF_45)) |
| bLH015 | *yukE::erm-Pyuk* | This work |
| bLH018 | *yukEDCBA::erm-Pyuk* | This work |
| bLH027 | *amyE::Phyperspank-lacZ (spec)* | RL2508 (Gift of Losick Lab) |
| bLH077 | *yukF::erm* | This work |
| bLH107 | *yukEDCBAyueB::erm* | This work |
| bLH110 | *yukBA::erm-Pyuk* | This work |
| bLH404 | *yukBA::erm-Pyuk; amyE::Phyperspank-yukBA-myc (spec)* | This work |
| bLH421 | *yukD::erm-Pyuk* | This work |
| bLH422 | *yukC::erm-Pyuk* | This work |
| bLH458 | *yukD::erm-Pyuk; amyE::Phyperspank-yukD-myc (spec)* | This work |
| bLH500 | *yukC::erm-Pyuk; amyE::Phyperspank-yukC-myc (spec)* | This work |
| bLH533 | *yukE::erm-Pyuk; amyE::Phyperspank-yukE (spec)* | This work |
| bLH579 | *yueB::erm-Pyuk* | This work |
| bLH581 | *yueC::erm-Pyuk* | This work |
| bLH585 | *yueD::erm* | This work |
| bLH589 | *yueB::erm-Pyuk; amyE::Phyperspank-yueB-HA (spec)* | This work |
| bLH590 | *yueB::erm-Pyuk; amyE::Phyperspank-yueB (spec)* | This work |
| bLH591 | *yueC::erm-Pyuk; amyE::Phyperspank-yueC-myc (spec)* | This work |
| bLH593 | *yueD::erm; amyE::Phyperspank-yueD-myc (spec)* | This work |
