# Supplementary material for: The ESX System in Bacillus subtilis Mediates Protein Secretion
Source: PLoS One. 2014 May 5;9(5):e96267. doi: 10.1371/journal.pone.0096267 (PMC4010439; doi:10.1371/journal.pone.0096267)
Supplement: Table S2 — Oligos used in this study. (DOCX) [file pone.0096267.s007.docx]

**Table S2. Oligos used in this study**

| **Primer** | **Sequence*** |
| --- | --- |
| oLH013 | CTTCTGCAGcagtacagtgaagtcatcagcac |
| oLH014 | CGGGTCGACacgttatttgtcatcttttttctc |
| oLH017 | GTACGGCCGctgatttgaagtttgagaagcttg |
| oLH018 | GTAGTCGACgattacaccgccaaatg |
| oLH019 | GTAGGATCCtcatccccttccttttattatg |
| oLH020 | GCTGAATTCacttatttttagaacgtcattattataaaaac |
| oLH021 | CTAGCATGCacttatttttagaacgtcattattataaaaac |
| oLH022 | GCTGAATTCtctatgtaagggtgatcctaaatgtatattg |
| oLH023 | GCATCTAGAttcagcctcgacagattgtg |
| oLH024 | GTTGAATTCattatgaaaggatctggttgaatg |
| oLH025 | CGTTCTAGAaatcatcataggattcagcatc |
| oLH026 | CTTGAATTCacatgaagcgcaggtcatc |
| oLH027 | GGTTCTAGAcgtcaccctcatttgcg |
| oLH028 | GATGCATGCtgctgaaagggagagtgctag |
| oLH029 | CGGTCTAGAcatgacattctggaattcagattc |
| oLH030 | CATGAATTCtctcatccccttccttttattatg |
| oLH031 | CTAGGATCCagcctaaatatgggctaattcac |
| oLH033 | GTACGGCCGcgcaacctctgacattgg |
| oLH034 | CTCGTCGACtttcttaatcaaacaaagaattttcc |
| oLH035 | GCATCTAGAggctaattcacttatttttagaacgtc |
| oLH044 | CGCAAGCTTACATAAGGAGGAACTACTATGTATATTGATATTACAATAG |
| oLH045 | GCGGCTAGCTCA*CAAGTCCTCTTCAGAAATGAGCTTTTGCT*CTAATATTTCAAGCCG |
| oLH052 | CTTGAATTCgcagcatggaaaagg |
| oLH053 | GCATCTAGAcgccttcttgttttcctgc |
| oLH067 | AGATAACATATGgcaggattaattcgtgtcac |
| oLH068 | ATTGGATCCatactgttatccgcggatttg |
| oLH097 | GTACGGCGGgattattcggagaatgaagag |
| oLH098 | CTTGTCGACgatcacccttacatagaaagagc |
| oLH099 | GTTCGGCGGtaattcgtgtcacacccg |
| oLH100 | CATGTCGACagatcctttcataatatttcaagc |
| oLH157 | CGCAAGCTTACATAAGGAGGAACTACTATGGCAGGATTAATTCGT |
| oLH158 | GCGGCTAGCTTATCCGCGGATTTGATTTG |
| oLH159 | GCCGGCGTCGACACATAAGGAGGAACTACTATGTTGAGTCTATTATGGGTT |
| oLH160 | TATCGCGCTAGCTCA*CAAGTCCTCTTCAGAAATGAGCTTTTGCT*CTCTAGCACTCTCCCTTTC |
| oLH161 | GCCGGCGTCGACACATAAGGAGGAACTACTATGTCAGGTGAACAAAAATCA |
| oLH162 | CGCGCTAGCTCA*CAAGTCCTCTTCAGAAATGAGCTTTTGCTCT*TTGTCATCTTTTTTCTC |
| oLH163 | GTACGGCCGctgtttgactcgattgaccg |
| oLH164 | CGGGTCGACtagcactctccctttcagcag |
| oLH165 | GATGAATTCgaaaaggggatcatgatttgtatg |
| oLH166 | CGCTCTAGAccgttattattgtacggcacc |
| oLH167 | GTACGGCCGtgatcagcagtctgttaatcgg |
| oLH168 | CGGGTCGACaaatcatgatccccttttcc |
| oLH169 | GATGAATTCtaacggaaaaggaggtttcc |
| oLH170 | CGCTCTAGAcgtaaacatgtcgagccc |
| oLH172 | GTACGGCCGcataccctctgcagcgg |
| oLH173 | CGGGTCGACtggaaacctccttttccg |
| oLH174 | GATGGATCCagcagatattttctgctcttttttttatac |
| oLH175 | CGCTCTAGAgcaaaatggctagtgcaatc |
| oLH176 | GCCGGCGTCGACACATAAGGAGGAACTACTATGCTCTCCTTTTCCCTTTTCATACC |
| oLH177 | TATCGCGCATGCTCA*CAAGTCCTCTTCAGAAATGAGCTTTTGCTC*CGCCTTCTTGTTTTCCTGCGC |
| oLH178 | GCCGGCGTCGACACATAAGGAGGAACTACTATGGAACTTTATATCATCACCGGAGCG |
| oLH179 | TATCGCGCATGCTCA*CAAGTCCTCTTCAGAAATGAGCTTTTGCTC*CAAAAACTCTTTAATATCATAAATGCGGCCG |
| oLH186 | CGGCGTCGACACATAAGGAGGAACTACTATGACAGAACAACGAAAAAGC |
| oLH187 | CGCGCATGCTCAAGCGTAGTCTGGGACGTCGTATGGGTACGCTTCATACGTTTCATC |
| oLH188 | GATGAAACGTATGAAGCGTAGCCATACGACGTCCCAGAC |
| oLH189 | GTCTGGGACGTCGTATGGCTACGCTTCATACGTTTCATC |

* Restriction sites underlined, myc or HA sequence italicized
