# Supplementary material for: The ESX System in Bacillus subtilis Mediates Protein Secretion
Source: PLoS One. 2014 May 5;9(5):e96267. doi: 10.1371/journal.pone.0096267 (PMC4010439; doi:10.1371/journal.pone.0096267)
Supplement: Text S1 — (DOCX) [file pone.0096267.s008.docx]

**Text S1**

**Strain construction:**

Note: All strains below are isogenic with PY79, unless otherwise listed.

**bLH015** [*yukE::erm-Pyuk*] was generated by a double crossover of pLH018 into competent bLH014 (*yukE::cat*) selecting for MLS(R) and screening for Cm(S). The double crossover was confirmed by PCR from genomic DNA.

**bLH018** [*yukEDCBA::erm-Pyuk*] was generated by a double crossover of pLH021 into competent bLH014 (*yukE::cat*) selecting for MLS(R) and screening for Cm(S). The double crossover was confirmed by PCR from genomic DNA.

**bLH019** [*amyE::Pyuk-lacZ (spec)*] was generated by a double crossover of pLH022 into competent PY79 selecting for Spec(R). The double crossover was confirmed by PCR from genomic DNA.

**bLH021** [*ΩPyuk-lacZ (cat)*] was generated by a single crossover of pLH024 into competent PY79 selecting for Cm(R).

**bLH077** [*yukF::erm*] was generated by a double crossover of pLH028 into competent PY79 selecting for MLS(R). The double crossover was confirmed by PCR from genomic DNA.

**bLH107** [*yukEDCBAyueB::erm*] was generated by a double crossover of pLH038 into competent PY79 selecting for MLS(R). The double crossover was confirmed by PCR from genomic DNA. There was no promoter inserted after the erm resistance cassette so there is a predicted polar effect on *yueCD* as well.

**bLH110** [*yukBA::erm-Pyuk*] was generated by a double crossover of pLH026 into competent PY79 selecting for MLS(R). The double crossover was confirmed by PCR from genomic DNA.

**bLH404** [*yukBA::erm-Pyuk; amyE::Phyperspank-yukBA-myc* (spec)] was generated by transforming genomic DNA from bLH266 (*amyE::Phyperspank-yukBA-myc (spec)*) into competent bLH110 selecting for Spec(R) and screening for MLS(R) and the inability to catabolize starch (*amyE-).* bLH266 was generated by a double crossover of pLH042 into competent bLH049 (*amyE::kan*) selecting for Spec(R) and screening for Kan(S) and the inability to catabolize starch (*amyE-*).

**bLH421** [*yukD::erm-Pyuk*] was generated by a double crossover of pLH066 into competent PY79 selecting for MLS(R). The double crossover was confirmed by PCR from genomic DNA.

**bLH422** [*yukC::erm-Pyuk*] was generated by a double crossover of pLH068 into competent PY79 selecting for MLS(R). The double crossover was confirmed by PCR from genomic DNA.

**bLH458** [*yukD::erm-Pyuk; amyE::Phyperspank-yukD-myc (spec)*] was generated by transforming genomic DNA from bLH052 (*amyE::Phyperspank-yukD-myc (spec))* into competent bLH421 selecting for Spec(R) and screening for MLS(R). bLH052 was generated by a double crossover of pLH034 into competent bLH049 (*amyE::kan*) selecting for Spec(R) and screening for Kan(S) and the inability to catabolize starch (*amyE-*).

**bLH500** [*yukC::erm-Pyuk; amyE::Phyperspank-yukC-myc (spec)*] was generated by transforming genomic DNA from bLH499 (*amyE::Phyperspank-yukC-myc (spec))* into competent bLH422 selecting for Spec(R) and screening for MLS(R). bLH499 was generated by a double crossover of pLH094 into competent bLH049 (*amyE::kan*) selecting for Spec(R) and screening for Kan(S) and the inability to catabolize starch (*amyE-*).

**bLH533** [*yukE::erm-Pyuk; amyE::Phyperspank-yukE (spec)*] was generated by transforming genomic DNA from bLH530 (*amyE::Phyperspank-yukE (spec))* into competent bLH015 selecting for Spec(R) and screening for MLS(R). bLH530 was generated by a double crossover of pLH107 into competent bLH049 (*amyE::kan*) selecting for Spec(R) and screening for Kan(S) and the inability to catabolize starch (*amyE-*).

**bLH579** [*yueB::erm-Pyuk*] was generated by a double crossover of pLH109 into competent PY79 selecting for MLS(R). The double crossover was confirmed by PCR from genomic DNA.

**bLH581** [*yueC::erm-Pyuk*] was generated by a double crossover of pLH111 into competent PY79 selecting for MLS(R). The double crossover was confirmed by PCR from genomic DNA.

**bLH585** [*yueD::erm*] was generated by a double crossover of pLH115 into competent PY79 selecting for MLS(R). The double crossover was confirmed by PCR from genomic DNA.

**bLH589** [*yueB::erm-Pyuk; amyE::Phyperspank-yueB-HA (spec)*] was generated by transforming genomic DNA from bLH444 (*amyE::Phyperspank-yueB-HA (spec))* into competent bLH579 selecting for Spec(R) and screening for MLS(R). bLH444 was generated by a double crossover of pLH095 into competent bLH049 (*amyE::kan*) selecting for Spec(R) and screening for Kan(S) and the inability to catabolize starch (*amyE-*).

**bLH590** [*yueB::erm-Pyuk; amyE::Phyperspank-yueB (spec)*] was generated by transforming genomic DNA from bLH501 (*amyE::Phyperspank-yueB (spec))* into competent bLH579 selecting for Spec(R) and screening for MLS(R). bLH501 was generated by a double crossover of pLH133 into competent bLH049 (*amyE::kan*) selecting for Spec(R) and screening for Kan(S) and the inability to catabolize starch (*amyE-*).

**bLH591** [*yueC::erm-Pyuk; amyE::Phyperspank-yueC-myc (spec)*] was generated by transforming genomic DNA from bLH586 (*amyE::Phyperspank-yueC-myc (spec))* into competent bLH581 selecting for Spec(R) and screening for MLS(R). bLH586 was generated by a double crossover of pLH116 into competent bLH049 (*amyE::kan*) selecting for Spec(R) and screening for Kan(S) and the inability to catabolize starch (*amyE-*).

**bLH593** [*yueD::erm; amyE::Phyperspank-yueD-myc (spec)*] was generated by transforming genomic DNA from bLH587 (*amyE::Phyperspank-yueD-myc (spec))* into competent bLH585 selecting for Spec(R) and screening for MLS(R). bLH587 was generated by a double crossover of pLH117 into competent bLH049 (*amyE::kan*) selecting for Spec(R) and screening for Kan(S) and the inability to catabolize starch (*amyE-*).

**Plasmid Construction:**

Note: All PCRs were conducted using PY79 genomic DNA as the template DNA.

**pLH018** [*yukE::erm-Pyuk*] was generated by replacing the *cat* cassette in pKM074 with an *erm* cassette from pBB286 (BamHI to SalI subcloning). A 500 bp region of DNA upstream of *yukE* (PCR using oLH017/oLH018) and a 500 bp region of DNA downstream of *yukE* (PCR using oLH022/oLH023) were inserted into the double-crossover integration vector pKM074. A 417 bp region of DNA between *yukF and yukE* was used as the *yuk* promoter (PCR using oLH019/oLH020) and inserted in front of the oLH022/oLH023 PCR product. pKM074 was a gift from D. Rudner.

**pLH021** [*yukEDCBA::erm-Pyuk*] was generated by replacing the *cat* cassette in pKM074 with an *erm* cassette from pBB286 (BamHI to SalI subcloning). A 500 bp region of DNA upstream of *yukE* (PCR using oLH017/oLH018) and a 500 bp region of DNA downstream of *yukBA* (PCR using oLH028/oLH029) were inserted into the double-crossover integration vector. A 417 bp region of DNA between *yukF and yukE* was used as the *yuk* promoter (PCR using oLH019/oLH021) and inserted in front of the oLH028/oLH029 PCR product.

**pLH022** [*amyE::Pyuk-lacZ (spec)*] was generated by inserting the 417 base pair stretch of DNA between *yukE* and *yukF* as the *Pyuk* promoter sequence (PCR of oLH030/oLH031) into the vector pDG1728 (*amyE::lacZ (spec))* ([46](#_ENREF_46)).

**pLH024** [*ΩPyuk-lacZ (cat)*] was generated by inserting the 417 base pair stretch of DNA between *yukE* and *yukF* as the *Pyuk* promoter sequence (PCR of oLH030/oLH031) into the Campbell *lacZ* integration vector pAVB161. pAVB161 was a gift from the Losick Lab.

**pLH026** [*yukBA::erm-Pyuk*] was generated by replacing the *cat* cassette in pKM074 with an *erm* cassette from pBB286 (BamHI to SalI subcloning). A 500 bp region of DNA upstream of *yukBA* (PCR using oLH013/oLH014) and a 500 bp region of DNA downstream of *yukBA* (PCR using oLH028/oLH029) were inserted into the double-crossover integration vector. A 417 bp region of DNA between *yukF and yukE* was used as the *yuk* promoter (PCR using oLH019/oLH021) and inserted in front of the oLH028/oLH029 PCR product.

**pLH028** [*yukF::erm*] was generated by replacing the *cat* cassette in pKM074 with an *erm* cassette from pBB286 (BamHI to SalI subcloning). A 500 bp region of DNA upstream of *yukF* (PCR using oLH033/oLH034) and a 500 bp region of DNA downstream of *yukF* (PCR using oLH019/oLH035) were inserted into the double-crossover integration vector.

**pLH034** [*amyE::Phyperspank-yukD-myc (spec)*] was generated by inserting the *yukD-myc* sequence (PCR using oLH044/oLH045) into the overexpression vector pDR111 (*amyE::Phyperspank (spec)*).

**pLH038** [*yukEDCBAyueB::erm*] was generated by inserting a 500 bp region of DNA upstream of *yukE* (PCR using oLH022/oLH023) and a 500 bp region of DNA downstream of *yueB* (PCR using oLH052/oLH053) were inserted into the double-crossover integration vector.

**pLH042** [*amyE::Phyperspank-yukBA-myc (spec)*] was generated by inserting the *yukBA-myc* sequence (PCR using oLH159/oLH160) into the overexpression vector pDR111 (*amyE::Phyperspank (spec)*). pDR111 was a gift from D. Rudner.

**pLH054** [*His6-SUMO-yukE*] was generated by inserting the *His6-SUMO-yukE* sequence into the pMAL-C5e backbone (New England Biolabs). His6-SUMO was subcloned between SphI and NdeI and YukE (PCR using oLH067/oLH068) was subcloned between NdeI and BamHI.

**pLH066** [*yukD::erm-Pyuk*] was generated by replacing the *cat* cassette in pKM074 with an *erm* cassette from pBB286 (BamHI to SalI subcloning). A 500 bp region of DNA upstream of *yukD* (PCR using oLH097/oLH098) and a 500 bp region of DNA downstream of *yukD* (PCR using oLH024/oLH025) were inserted into the double-crossover integration vector. A 417 bp region of DNA between *yukF and yukE* was used as the *yuk* promoter (PCR using oLH019/oLH020) and inserted in front of the oLH024/oLH025 PCR product.

**pLH068** [*yukC::erm-Pyuk*] was generated by replacing the *cat* cassette in pKM074 with an *erm* cassette from pBB286 (BamHI to SalI subcloning). A 500 bp region of DNA upstream of *yukC* (PCR using oLH099/oLH0100) and a 500 bp region of DNA downstream of *yukC* (PCR using oLH026/oLH027) were inserted into the double-crossover integration vector. A 417 bp region of DNA between *yukF and yukE* was used as the *yuk* promoter (PCR using oLH019/oLH020) and inserted in front of the oLH026/oLH027 PCR product.

**pLH094** [*amyE::Phyperspank-yukC-myc (spec)*] was generated by inserting the *yukC-myc* sequence (PCR using oLH161/oLH162) into the overexpression vector pDR111 (*amyE::Phyperspank (spec)*).

**pLH095** [*amyE::Phyperspank-yueB-HA (spec)*] was generated by inserting the *yueB-HA* sequence (PCR using oLH186/oLH187) into the overexpression vector pDR111 (*amyE::Phyperspank (spec)*).

**pLH107** [*amyE::Phyperspank-yukE (spec)*] was generated by inserting the *yukE* sequence (PCR using oLH157/oLH158) into the overexpression vector pDR111 (*amyE::Phyperspank (spec)*).

**pLH109** [*yueB::erm-Pyuk*] was generated by replacing the *cat* cassette in pKM074 with an *erm* cassette from pBB286 (BamHI to SalI subcloning). A 500 bp region of DNA upstream of *yueB* (PCR using oLH163/oLH0164) and a 500 bp region of DNA downstream of *yueB* (PCR using oLH165/oLH166) were inserted into the double-crossover integration vector. A 417 bp region of DNA between *yukF and yukE* was used as the *yuk* promoter (PCR using oLH019/oLH020) and inserted in front of the oLH165/oLH166 PCR product.

**pLH111** [*yueC::erm-Pyuk*] was generated by replacing the *cat* cassette in pKM074 with an *erm* cassette from pBB286 (BamHI to SalI subcloning). A 500 bp region of DNA upstream of *yueC* (PCR using oLH167/oLH0168) and a 500 bp region of DNA downstream of *yueC* (PCR using oLH169/oLH170) were inserted into the double-crossover integration vector. A 417 bp region of DNA between *yukF and yukE* was used as the *yuk* promoter (PCR using oLH019/oLH020) and inserted in front of the oLH169/oLH170 PCR product.

**pLH115** [*yueD::erm*] was generated by replacing the *cat* cassette in pKM074 with an *erm* cassette from pBB286 (BamHI to SalI subcloning). A 500 bp region of DNA upstream of *yueD* (PCR using oLH172/oLH0173) and a 500 bp region of DNA downstream of *yueD* (PCR using oLH174/oLH175) were inserted into the double-crossover integration vector.

**pLH116** [*amyE::Phyperspank-yueC-myc (spec)]* was generated by inserting the *yueC-myc* sequence (PCR using oLH176/oLH177) into the overexpression vector pDR111 (*amyE::Phyperspank (spec)*).

**pLH117** [*amyE::Phyperspank-yueD-myc (spec)*] was generated by inserting the *yueC-myc* sequence (PCR using oLH178/oLH179) into the overexpression vector pDR111 (*amyE::Phyperspank (spec)*).

**pLH133** [*amyE::Phyperspank-yueB (spec)*] was generated by introducing a STOP codon at the end of the *yueB* coding sequence in the plasmid pLH095 [*amyE::Phyperspank-yueB-HA (spec)*] using QuikChange site directed mutagenesis (Stratagene) (primers oLH188/oLH189).
